# Social factors determining maternal and neonatal mortality in South Africa: A qualitative study

**DOI:** 10.4102/curationis.v39i1.1571

**Published:** 2016-06-22

**Authors:** Rose M.M. Mmusi-Phetoe

**Affiliations:** 1 Department of Health Studies, University of South Africa, South Africa

## Abstract

**Background:**

South Africa’s maternal mortality ratio has increased from 150/100 000 in 1990 to 269/100 000 live births in 2015 against the Millennium Development Goals 5 (MDG5) target of 38/100 000, indicating slow progress in improving maternal health. The neonatal mortality rate was 14/1000 live births against the MDG4 target of 7/1000. The purpose of the article was to outline the socio-economic factors that determine maternal and neonatal mortality in South African communities.

**Objectives:**

To identify and describe the social determinants of maternal and neonatal mortality in South Africa.

**Method:**

A qualitative study using audio-taped individual interviews was conducted. The interviews included 10 pregnant women who were purposefully recruited from the antenatal clinic attendees in a public hospital. The interviews were conducted in isiZulu and later translated into English by the researcher who is fluent in both. Data were analysed using the World Health Organization’s (WHO) Commission on Social Determinants of Health framework.

**Results:**

Findings revealed that poverty was an underlying factor to the vulnerability to illness and death of the mothers and their neonates. Other determinants were found to be the nutritional inadequacies, neglect and abuse by male partners, HIV or AIDS, inattention to reproductive health and violation of reproductive rights, and powerlessness of women and health system issues such as poor quality and incompetent health care.

**Conclusion:**

It is apparent that poverty plays a major role in determining the health of mothers and neonates. This requires more coordinated multi-sectorial interventions to address both the social determinants and direct causes of maternal and neonatal deaths.

## Introduction

Maternal and child mortality remain a major challenge to the health systems worldwide. The global maternal mortality ratio (MMR) was estimated at 210/100 000 live births in 2013, down from 380/100 000 live births in 1990 (World Health Organization [WHO], United Nations Children’s Fund [UNICEF], United Nations Fund for Population Activities [UNFPA], the World Bank and the United Nations Population Division 2014). This was the period when many countries around the world committed to the Millennium Development Goals (MDGs). Data collected by the WHO reflect that more than half a million maternal deaths occur globally every year with 99% occurring in developing countries, many of which are in Africa, and only 1% occur in the developed countries (WHO, UNICEF, UNFPA, the World Bank and the United Nations Population Division 2014).

The global under-five mortality rates have also decreased from an estimated rate of 90/1000 live births in 1990 to 48/1000 live births in 2012 (UN Inter-agency Group for Child Mortality Estimation 2013; UNICEF, WHO, The World Bank and United Nations 2013). These reports note that all the regions, except sub-Saharan Africa and Oceania, have reduced their under-five mortality rate by 50% or more. Sub-Saharan Africa and South Asia remain the regions with the highest numbers of child deaths (UN Inter-agency Group for Child Mortality Estimation 2013).

As with most countries in sub-Saharan Africa, maternal and child deaths still present a significant burden in South Africa, indicating slow progress in improving maternal health, and the intimately linked perinatal and new-born health (The Partnership for Maternal, Newborn and Child Health 2015). Dorrington *et al.* (2014) estimated the South African MMR at 269/100 000 live births in 2010 whilst Solarin and Black (2013) estimated it to be 410/100 000 live births in that year. Although the estimation of the South African MMR shows inconsistency, the figures confirm that South Africa is unlikely to meet its MDG target of 38/100 000 live births by 2015. According to the WHO, of all the nine provinces of South Africa, KwaZulu Natal (KZN), a province where the study was conducted, has the highest MMR of 400/100 000 (South African Broadcasting Corporation [SABC] 4 April 2013).

South Africa was one of the 189 countries that endorsed the United Nations’ Millennium Declaration in 2000 (United Nations 2000), thus committing to meeting eight goals, referred to as the Millennium Development Goals (MDGs) by 2015, using 1990 as a base year (Statistics South Africa and National Treasury 2007). The MDG 5 called for countries to improve maternal health and reduce the MMR by three-quarters (Ronsmans & Graham 2006). MDG 4 called for a reduction in the child mortality rate by two-thirds (Statistics South Africa and National Treasury 2007). Chopra *et al.* (2009) confirm that maternal mortality and child mortality in South Africa have both increased since the MDG baseline of 1990.

The maternal mortality and child mortality rates are indicative of the health of the population and reflect deeper issues such as an inequitable distribution of the country’s resources, poverty, unemployment, social exclusion, deprivation, lack of access to quality public services and unmet women’s needs. Wuyts, Mackintosh & Hewitt (1997) made the observation that the health indicators, especially the MMR and child mortality rates are sensitive to deprivation, hardship and are a better reflection of the extent of satisfaction of basic needs. The high rates of both MMR and NMR in KZN prompted the researcher to carry out the study.

### Problem statement

South Africa continues to experience high maternal and child mortality. However, the link between maternal and child morbidity and mortality and the vulnerability of women is often not sufficiently problematised and addressed by policy makers, resulting in ineffective plans of action. Attempts to address this problem often focused on finding solutions to complications related to pregnancy and childbirth through narrowly defined, ‘quick-fix’ medical-technical solutions, instead of focusing on the root causes of the persistently high levels of maternal and child morbidity and mortality. For example, the antenatal care coverage is 97% and skilled attendant deliveries 94% according to Solarin and Black (2013) and the Henry Kaiser Family Foundation (2015) respectively; however, the problem of persistently high maternal and newborn deaths remains. Little is documented on the socio-economic determinants of maternal and neonatal mortality.

The purpose of the article was to trace, from the women’s perspective, the underlying conditions that expose women and their neonates to the risk of ill-health and mortality in South African communities.

## Background

Socio-economic factors have been inseparably linked with the high burden of poverty-related diseases and the vulnerability of women (Goldwyer 2014). In South Africa, the roots of poverty and the associated maternal and child mortality are a result of the previous apartheid era. The majority of black people living in rural areas were mostly affected and suffered from massive malnutrition. Goldwyer (2014) highlights that infectious diseases and malnutrition which were eliminated in the white population destroyed the rural poor black population in the homelands with the resultant burden of infant and maternal mortality on this population group. During the same era, the education system was exclusive and black women were mostly affected (Sachs 2002, in Hallstrom, Guehlstorf & Parkes 2015). The majority of women remained under-educated and illiterate, which in turn affected women’s social positions and access to improved health.

Differences in mortality rates within the South African population groups remain (Statistics South Africa 2014). Studies conducted in 2002 reflect that the infant mortality rates varied between 7/1000 live births in the white population and 67/1000 live births in the black population (Coovadia *et al.* 2009). The differences were further evident between and within the provinces whereby in the year 2000, the under-five mortality ranged from 16/1000 live births in the Western Cape to 116/1000 live births in KZN and almost threefold higher in the squatter camps of Western Cape than in the metropolitan areas (Coovadia *et al.* 2009). Coovadia *et al.* (2009) highlight that the marked differences in the rates of diseases and deaths between the races clearly reflect the geographical, class, sexual and racial divisions in accessing the basic needs and other social determinants of health.

Coovadia *et al.* (2009:18) further made an observation on the HIV prevalence rates in South Africa showing that white and Indian men and women have lower prevalence rates of HIV (0.6% and 1.9% respectively), whilst higher rates of 13.3% were reported in the black population. According to Coovadia *et al.* (2009) white females enjoyed a 50% longer life expectancy rate compared to their black counterparts. Sachs (2002) asserts that the spread of HIV/AIDS in South Africa today could be attributed to the residuals of the apartheid system.

## Aim of the article

The aim of the article was to explore the social factors determining maternal and neonatal mortality in South Africa.

## The significance of the article

Maternal and child mortality remain a key indicator of the general capability of the health system to deliver preventive, promotive and curative health services in South African. The social determinants of maternal and child mortality provide an illustration of the shortcomings in the current curative based approaches and interventions. The article exposes the main inhibiting social conditions amongst the most affected, namely black, poor and rural women, which, if addressed, would possibly lead to a decline of the consistently high rates of maternal and child mortality in South Africa.

## Research methods and design

### Research design

This was a qualitative study that used a descriptive design.

### Conceptual framework

**FIGURE 1:**
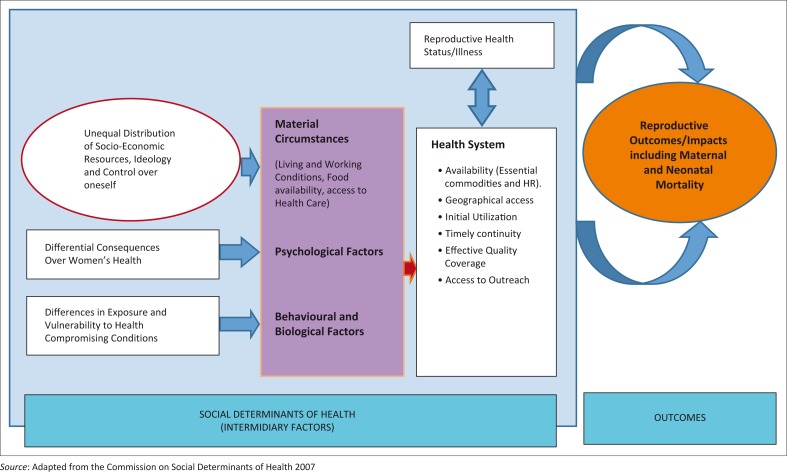
The Analytic Framework of the Social Determinants of Health in Relation To Maternal and Neonatal Mortality.

The Social Determinants of Health (SDH) framework developed by the WHO’s commission in 2007 was used as the study framework (Figure 1). The framework provided a way to illustrate pathways by which social determinants affect reproductive health outcomes, including maternal and neonatal morbidity and mortality, given the increasing evidence of significant social stratification in the health status. Any effort to reduce maternal and neonatal illness and deaths must operate through these events. If these are addressed, the risk to maternal and neonatal mortality should decline. A summary of the interaction of social determinants of health and maternal and neonatal mortality is presented in Figure 1.

### Setting and sample

The study took place in 2011 at a public hospital in KZN, South Africa. It is the second most populous province in South Africa, covering a total area of 92 100 sq km of the total land surface of South Africa (Health Systems Trust [HST] 2008). Slightly half of the population (49.5%) in the KZN province lived on less than $2.00 a day (approximately R15.60 a day) in 2010 (South African Institute for Race Relations [SAIRR] 2011). Its population was 5 162 815, the highest number of all the South African provinces, many living in poverty. Of these 5 044 217 were black people compared to only 3336 white people (SAIRR 2011).

KZN has been organised into 11 health districts, amongst which is iLembe ([KZN] Department of Health 2015), a district chosen for the study site. The District Health Plan 2014/15 in KZN (2015) highlighted that the iLembe district has a total population of 630 464, and incorporates four sub-districts, namely Ndwedwe (134 56), Maphumulo (99 213), Mandeni (143, 586), and KwaDukuza (242 502).

iLembe was classified by the District Health Barometer 2007/08 as one of the districts that are socially deprived in KZN (HST 2008:18). Of all the districts of KwaZulu-Natal, iLembe has been mostly affected by the HIV/AIDS epidemic with an HIV prevalence rate of 42.5% amongst the ANC women in 2010 (South African Department of Health [DoH] 2010). At the time of the study, iLembe was identified as one of the National Department of Health’s 18 priority districts that required acceleration of proven, low-cost but high-impact maternal, neonatal, child and women’s health and nutrition interventions (DoH 2008).

The only public hospital in iLembe district from which data were collected, serves most of the nearby rural communities in the surrounding areas. The hospital has various maternal and child-care programmes, which made it possible to collect data to inform the study.

### Sample

A volunteer sample of 10 women was purposively recruited for the study from the antenatal care units (ANC) of a study hospital in KZN. Informed by literature about the most affected women by high MMR in this country and the researcher’s work experience as a maternal and child survival specialist, the inclusion criteria for the women were that they had to be pregnant, black and residing in a rural area in South Africa and be dependent on public health care. In addition, the women who were characterised by their encounter with adverse reproductive conditions such as early/teenage pregnancy, excess fertility, closely-spaced births, child-bearing above age 35 years, and HIV/AIDS infection were to be included in the sample.

Recruitment of volunteer interviewees meeting the inclusion criteria was carried out in consultation with the staff of the study hospital, after obtaining approval to conduct a study from the hospital management. A professional nurse in charge of reproductive units was delegated to support the researcher with the recruitment of interviewees. The researcher was then allocated a research site at the ANC which was quiet, private and convenient for the interviews. The professional nurses assisted the researcher with identifying the women as they were queuing up and receiving the ANC services. The women were then purposefully selected by the researcher to ensure adequate representation according to the sample and sub-samples of the study. The researcher was further advised of the weekly days to visit the ANC, thus finding and interviewing different categories of women as per the study to increase adequate representation. Table 1 reflects the sub-samples of the main study sample of 10 women who were purposefully selected in the study.

**TABLE 1:** Study population by sub-samples.

Sub-Sample	Main features of the sub-sample
1. Women in the study	**Teenaged interviewees:** women younger than 18 years who had given birth to live children in the last two years and were pregnant
	**Interviewees older than 35 years:** women at the lower end of the reproductive age group and were pregnant
	**Interviewees with closely spaced births:** women between the ages of 18 and 35 years who have had live births (excluding multiple births) spaced 24 months or less over their lifetimes and were pregnant
	**Interviewees with high parities:** women who have had five or more live births over their lifetime and were pregnant
	**HIV–positive Interviewees:** women who were HIV-positive and pregnant at the time of the study

*Source*: Adapted from Mmusi-Phetoe 2011

### Data collection

Data were collected over a period of over four weeks. In-depth face-to-face interviews were conducted with 10 pregnant women attending the ante-natal clinic at one of the villages in one of the districts of KZN. The individual interviews were conducted in a private room allocated to the researcher. The guide that was structured to solicit the social determinants of maternal and neonatal morbidity and mortality from women was used. Interviews were audio-taped with the participants’ permission. Issues of confidentiality, anonymity and the right to withdraw from the study were continuously emphasised.

Interviews were conducted in the participants’ language, IsiZulu, and later translated into English by the researcher and the linguist in both languages from the University of South Africa (UNISA).

### Data analysis

The SDH framework developed by the WHO’s Commission on Social Determinants of Health (CSDH) (2007) was used as a guide in the thematic analysis of the data. The SDH framework holds that medical-technical solutions are important but not sufficient interventions to improve the health status of a population (CSDH 2007). That means, beyond curative based interventions, an action on the social factors can contribute to improving the health status of a population.

In keeping with Colaizzi’s (1978) seven steps of data analysis, the researcher performed thematic analysis as follows:

- Reading and re-reading the transcripts in order to obtain a general sense from the women’s narratives about the social factors that affect their health and determine maternal and neonatal mortality.
- Extracting significant statements from the transcripts that relate to the basis of the study.
- From the statements, formulating meaning in relation to the premise of the study.
- Organising formulated meaning into clusters which allow themes to emerge.
- Integrating themes into all-inclusive description of the participants’ statements.
- Identifying and providing a comprehensive description of the social factors that determine maternal and neonatal deaths.
- Validating the findings by cross-checking with the research participants if their responses were all captured to which they agreed.

## Ethical considerations

Ethical clearance was obtained from the ethics committee of the University of South Africa (UNISA) prior to conducting the study. The aim of the study was explained to the research participants and they voluntarily agreed to participate. The consent to participate in the study was obtained only after the researcher had disclosed all other relevant information to the prospective participants. The research participants were given an opportunity to choose what should and should not happen to them during the interviews, by signing the pre-prepared consent forms.

Confidentiality was maintained by using participants’ numbers and not their names. The room used for the interviews is usually the one used for counselling and allow for privacy. Participants were free to withdraw from the study at any time they wished to and that was emphasised throughout the study.

All other universal ethical principles related to human subjects’ research were observed.

## Results and discussion

Four key themes emerged in the final step of the data analysis, namely, nutritional inadequacies, neglect by male partners, pregnancy, and abuse. These were cited by the women who were interviewed as the main social factors that determined maternal and neonatal mortality.

### Demographic details of women

All 10 participants were women in the reproductive age groups of 15 to 49 years. Table 2 depicts the demographic characteristics of the participants.

**TABLE 2:** Demographic characteristics of the participants.

Demographic Variables	Number
**Ages**	
15-19	2
20-24	2
25-29	2
30-34	1
35-39	2
40-44	1
45-49	0

**Total**	**10**
**Educational Attainment**	
None	1
Grades 1-4	1
Grades 5-8	3
Grades 9-12	5
Tertiary education	0

**Total**	**10**
**Age at first birth**	
15-19	6
20-24	3
25-29	1
30-34	0
35-39	0
40-44	0
45-49	0

**Total**	**10**
**Marital Status**	
Single	8
Customary marriage	0
Civil Marriage	0
Cohabiting	2

**Total**	**10**
**Number of live births**	
0-2	2
04-Mar	6
5+	2

**Total**	**10**
**Employment Status**	
Unemployed	7
Employed	2
Other: e.g. self-employed as petty trader	1

**Total**	**10**

*Source*: Adapted from Mmusi-Phetoe 2011

Five of the ten participants had grade 9 to 12 and none had a tertiary education. The age range of the participants was 15 to 40 years. Only one was married. The nine unmarried women were single. The number of pregnancies was between one and nine.

### Themes generated from the study

The four major themes that emerged from the women’s transcribed interviews as important social factors determining maternal and neonatal morbidity and mortality are reflected in Table 3.

**TABLE 3:** Themes and categories generated from the study.

Theme	Number of women mentioning the theme
Nutrition	5
Neglect by male partners	3
Pregnancy itself	3
Abuse	3

*Source*: Adapted from Mmusi-Phetoe 2011

### Nutrition

Lack of adequate nutrition emerged a major theme contributing to increased morbidity and mortality. The extracts below testify inadequate nutrition.

‘One has to be HIV-positive to receive assistance from the government; otherwise no one cares for women if they are not HIV-positive, regardless of how poor they may be. The health professionals advise us that we should eat healthy; they do not have any idea if we have the food that they tell us to eat.’ (P1:35yrs)

‘Pregnancy used to be something that our ancestors used to be pleased about, but now things have changed. Once a woman gets pregnant, the community and the family worry whether a woman will carry a baby till delivery. Our bodies are already weak from the kind of food we eat which is not good for a pregnant woman. We are truly starving. A baby is an additional mouth to feed. Right now I am pregnant and I can’t feed myself. How am I going to feed this child (pointing at the abdomen). I am not working, my mother is the only one working to feed the family. We are seven in the house. We do not eat during the day. We eat only in the evening when everyone is home, mostly porridge and sour milk (amasi) or pap and chicken livers or pap and achaar … the kind of food we eat which is not good for a pregnant woman.’ (P2:38yrs)

### Neglect by male partners

Neglect and abandonment by male partners was perceived as a second important determinant of poor health outcomes for women in South Africa. Abandonment by a partner, especially when pregnant, was narrated as a deeply degrading and humiliating experience for women, causing serious ill health. Some interviewees recounted in the following extracts:

‘To tell the honest truth, men do not love us. All they want is to sleep with us; when we fall pregnant,they run away. One becomes a laughing stock in the community and one feels so dirty. This too causes such an embarrassment to our parents. One neighbour commented sarcastically that some girls are just objects that men relieve themselves with sexually when they have lust.’ (P3:21yrs)

‘I have come to realise that we – the young ones – fall in love with older men who are already married. They manipulate us so that we can have sex with them and when we fall pregnant they run away. Men also do not want to carry the responsibility of looking after their offspring. What I mean is that they do not want to maintain their offspring.’ (P4:20yrs).

### Pregnancy itself

The chances of the mother suffering from maternal morbidity increase at the time of pregnancy as confirmed in the following extracts:

‘With the previous pregnancies of my other two children, I was hospitalised for shortage of blood and received blood. However, for this current pregnancy I was hospitalised for stomach cramps. I also had high blood pressure. I actually slept at the hospital before delivery of my two other children and after delivery, as I always deliver through Caesarean sections.’ (P5:22yrs)

She continued:

‘You see (addressing the researcher), right now I am so ill from this pregnancy. Even with my previous, other pregnancies I was ill and was admitted to the hospital with shortage of blood, swelling and high blood pressure. Now this time I am bleeding. The doctor strongly warned me against another pregnancy because pregnancy affects my whole body. I am now suffering from a heart (problem). The doctor advised me that I should close (referring here to female sterilisation via tubal ligation), because I will die if I dare fall pregnant again. Even now I am scared that I will die and leave my children.’ (P5:22yrs)

‘I had high blood pressure with all my previous pregnancies. Then I would always be given tablets and attend my clinics. I’ve been requested to be managed at the hospital and not the nearby clinics like other women. Even before I went for my booking, on the 10th of January, I went to the doctor first so that he could tell me how many months pregnant I was. Then I went for my first booking at the clinic but was then referred to the hospital. All the tests were done … I was advised to go for an ultrasound on the 14th so that I can know exactly how far pregnant I am. I will be given dates for attending check-ups before I go for delivery at the Hospital … I delivered child number 4 through Caesarean section at the Hospital, because I was bleeding vaginally. I really do not know why I was bleeding; hence I am so scared about what will happen to me or my baby during the time of delivery.’ (P6:38yrs).

### Abuse

All participants mentioned being either physical, emotionally or financially abused.

The following extracts confirm abuse:

‘I do not know what I have done to God. I have been pregnant nine times. I was never married. I had a partner who was the father of my other children. He was abusing me physically until I left him. I left him with my other children. One of my children died, but he never even told me. I picked it up on the street that he had died …Now I have this partner who visits me most of the time. He is aware that I am pregnant but most of the time he does not buy food. I have to feed him. I am struggling … I went to the clinic for my first (antenatal care) booking only today, although I am already 9 months pregnant. I didn’t have money to go for my booking. I was then told that I am HIV-positive. This man gave me HIV because he runs around with other women. The nurses asked me to tell him to also go for the test. I will not tell him that I am HIV-positive because he will beat me up. I want him to go to the clinic, get tested and get the results for himself. He must feel it too.’ (P7:40yrs)

‘The father of my second child deserted me when I was nine months pregnant. He has never seen his child and the child is already two years old. Throughout pregnancy he beat me, forcing me to sleep at his place so that he can have sex with me, when he knew that he didn’t love me. He was just using me.’ (P4:20yrs)

## Trustworthiness

To ensure credibility of the article, the researcher stayed in the study hospital for two weeks prior to data collection. The researcher further used member checking by asking the participants if what she has written is what they had said. This was further corroborated by an expert in qualitative research who read the transcripts independently and affirmed findings.

## Discussion

The social factors that determine maternal and neonatal morbidity and mortality were examined by means of in-depth interviews with the pregnant women attending ANC at one district in KZN. Malnutrition stood out as a social root and factor determining morbidity and increased mortality of women in KZN. All 10 women reported a lack of food and nutritious foodstuffs as subjecting them to the risk of ill-health. Although starchy foodstuffs were available (albeit in inadequate quantities), protein-rich foodstuffs were reported as being scarce and unaffordable. However, a focus on inadequate nutrition as a cause of death obscures the full character of social problems suffered by black, poor, rural women.

Malnutrition is a symptom of deprivation related to poverty and norms regarding food distribution in the family or community. In this regard, addressing poverty seems a priority, as countries with greater income equity show better health outcomes, better educational performance of school children, lower teenage birth rates and reduced mortality rates (McKenzie & Pinger 2015).

Neglect by male partners as a social factor in determining maternal and neonatal mortality also stood out as the major concern by the women. According to the WHO (2013), having children is a partnership issue, and that women have a right to health, but protecting that right often depends on a partner’s support. The questions which arise are: why do men impregnate women and then disappear? Moreover, why is the apparent absence of responsible fatherhood so easily tolerated by the women? A possible explanation is that in a patriarchal society like that of the KZN, women’s sexuality is shaped in service of men’s needs and defined according to male norms.

Pregnancy itself emerged as one of the main factors of ill health and death; however, some contradictions were also noticed during the period of data collection. For example, almost all the women were found to be doing very little to prevent unplanned pregnancies. Problems with unmet need because of inaccessibility of the services and not having money for transport to go to family planning clinic were voiced in the study. These indirectly exposed women to the risk of ill-health as a result of unplanned pregnancy.

Of the five women who were allegedly physically abused, four were abused by boyfriends. One had been beaten up by her brother because of her pregnancy. Material dependency, craving for emotional affection, lack of alternative opportunities and culturally sanctioned female subordination to the socially constructed values and norms leave few options for a poor woman other than the physical and material protection from a man as a last resort.

Almost all of the women interviewed came from and still live under circumstances that could be described as destitution. Therefore, poverty amongst the women was found to be the underlying determinant and common denominator for all the medical, social and political factors.

## Limitations of the study

The findings of the article regarding the women are based on the views of women in one district only in KZN, and may not be applicable to other districts or other provinces. In addition, the study might have missed the views of women who did not attend the antenatal care unit during the period of study, precisely because of their social and economic circumstances.

## Recommendations

The following recommendations are made based on the study:

- The conception of maternal and neonatal morbidity and mortality has to change from the view that this phenomena is disease-specific thus warranting curative based interventions only. The nurses and other health professionals need to recognise that high rates of maternal and neonatal morbidity and mortality are a result of the social factors working through other paths and finally direct factors to cause ill-health, complications and deaths of women, before, during and after pregnancy. Therefore, interventions have to start by addressing the social factors determining maternal morbidity and mortality in order to reduce the vulnerability of women to illness and deaths. This will make an impact in reducing high rates of maternal and neonatal morbidity and mortality.
- In line with the above, the community health nurses should be empowered to identify conditions that expose women and their neonates to the risk of ill-health and mortality and take appropriate measures to address such conditions when identified. Further, the health officials need to focus beyond clinical setting when they attend to pregnant women during ante-natal clinic sessions in health centres.

## Conclusion

The article established that poverty was for women the root of ill health, manifesting itself in material lack and poor health. Women entered their reproductive years already disadvantaged. Therefore, poor maternal and neonatal health outcomes, poverty and household level explanatory variables influenced each other and produced certain values, culture and attitude to life, and interplay of risk variables during reproductive phases. The study aligns with the 1978 declaration of Alma Ata which put emphasis on the recognition of the importance of empowering individuals and communities, redressing inequalities in resource distribution and recognition of the real causes of ill-health.
